# Translational control of cyclins

**DOI:** 10.1186/1747-1028-6-5

**Published:** 2011-02-11

**Authors:** Woan-Yuh Tarn, Ming-Chih Lai

**Affiliations:** 1 Institute of Biomedical Sciences, Academia Sinica, 128 Academy Road Section 2, Nankang, Taipei 11529, Taiwan; 2 Department of Physiology, College of Medicine, National Cheng Kung University, 1 University Road, Tainan 70101, Taiwan

## Abstract

Regulation of cyclin levels is important for many cell cycle-related processes and can occur at several different steps of gene expression. Translational regulation of cyclins, which occurs by a variety of regulatory mechanisms, permits a prompt response to signal transduction pathways induced by environmental stimuli. This review will summarize translational control of cyclins and its influence on cell cycle progression.

## Review

The cell cycle is a fundamental and ordered process in which DNA is replicated and homologous chromosomes are segregated and equally distributed to daughter cells. The rate of protein synthesis oscillates during the cell cycle, indicating the importance of translational control for cell cycle progression [1]. Moreover, translational control allows rapid and reversible alterations of protein levels in response to various physiological and pathological conditions [2]. Therefore, it is important for us to have a comprehensive understanding of cell cycle-dependent translation control.

Prior to DNA synthesis, the cell cycle phase termed G1 is a period of cell growth and characterized by a high level of both protein synthesis and metabolic rate. During G1, cells also need to ensure their competency to undergo mitosis [3]. After passing through the G1/S checkpoint, cells enter S phase for DNA replication. However, most mammalian cells pause during G1 and enter a quiescent stage termed G0; certain cell types (e.g. neurons and muscle cells) may remain at this stage and undergo differentiation. Global protein synthesis is largely down-regulated in G0, but a subset of mRNAs is specifically translated to ensure cell survival [1]. At G2/M phase, ~60-80% of cap-dependent translation is inhibited whereas alternative mechanisms of translation may be activated for expression of certain mitotic factors [1].

Many key regulatory factors are expressed and activated at very specific points during the cell cycle. For example, the activity of cyclin-dependent kinases (Cdks) oscillates throughout the cell cycle and is essentially modulated by associated cyclins. The expression level of cyclins is primarily regulated by transcription of cyclin genes and turnover of cyclin proteins [4,5]. Over the past two decades, however, translation has also emerged as a key point at which the levels of cell cycle regulators are modulated. In this review, we discuss current knowledge on the translational control of cyclins.

## Translation initiation

Translation is essentially divided into three stages: initiation, elongation and termination. Eukaryotic translational control mainly occurs at the initiation step, which engages a large number of eukaryotic translation initiation factors (eIFs) and the ribosomal subunits [6,7]. In canonical cap-dependent translation initiation, the eIF4F complex, which is composed of the cap-binding protein eIF4E and two other initiation factors, eIF4G and eIF4A, binds to the 5'-end cap structure of mRNAs. eIF4G acts as a scaffold protein to mediate the interaction between eIF4E at the 5' end of mRNA and the poly(A) binding protein (PABP) that binds to the 3' poly(A) tail, thus circularizing the mRNA. Subsequently, the 43 S pre-initiation complex containing the 40 S ribosome, the eIF2-GTP-Met-tRNAi ternary complex and several initiation factors, joins eIF4F-bound mRNA and scans the mRNA for the AUG initiation codon. Some mRNAs harboring secondary structure or with a high GC content in the 5' untranslated region (UTR) may require additional *trans*-acting factors for ribosome scanning [8]. After initiation codon recognition, the 60 S ribosomal subunit joins to form the 80 S initiation complex.

## Cellular signaling pathways that affect translation and regulate cell cycle progression

**Figure 1:**
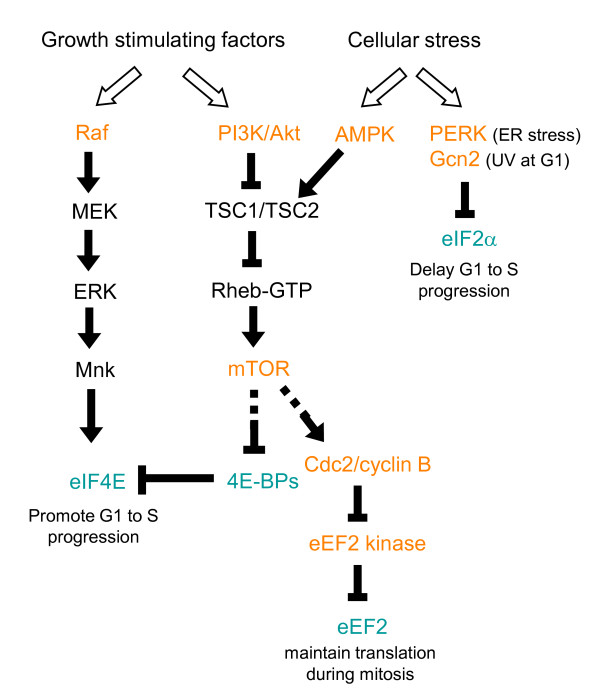
**Links between cellular signaling pathways and cell cycle control via translational regulation**. Cell growth stimulating factors activate the mTOR and Ras/Raf-MAPK signaling pathways. These two signaling cascades may regulate translation of cell cycle regulatory factors by modulating the activity of some translation factors, and thereby promote cell cycle progression and cell survival. Negative environmental factors may inhibit cell cycle also by targeting the translation factors. Different signaling pathways may have common targets to coordinate cell cycle regulation. Note that this simplified schematic diagram only illustrates the factors described in the text. Kinases and translation factors are labeled in orange and blue.

During the cell cycle, several cellular signaling pathways are induced and regulate cell cycle progression via control of the translation of cell cycle factors; the most important are the Akt/mammalian target of rapamycin (mTOR) and Ras/mitogen-activated protein kinase (MAPK) pathways [9] (Figure 1). A number of growth stimulating factors such as growth hormones, cytokines and nutrients initially activate the phosphoinositide 3-kinase (PI3K) and Akt kinase. PI3K/Akt signaling suppresses the activity of the Rheb GTPase activating complex (TSC1/TSC2) and thereby increases the level of GTP-bound Rheb, which in turn induces mTOR signaling [10]. mTOR signaling can target to several translation factors or regulators (see below for the detail). Activation of mTOR up-regulates the translation of key factors required for cell cycle progression from G1 to S phase, including specific G1/S cyclins, and thus promotes cell proliferation [11]. Inhibition of mTOR results in G1 arrest in some mammalian cells [1]. Moreover, mTOR signaling also promotes completion of the first mitotic division in sea urchin embryos by promoting cyclin B translation [12]. On the other hand, growth-inhibiting signals can activate the AMP-activated protein kinase (AMPK) that directly phosphorylates and activates TSC1/TSC2 and therefore causes mTOR inhibition [13].

Growth factors or cytokines can activate MAPKs such as p38 and extracellular signal-regulated kinase (ERK). These MAPKs subsequently activate distinct families of MAPK-activated protein kinases, such as Mnk1/2, that in turn directly phosphorylate certain translation factors. For example, phosphorylation of eIF4E by Mnk1/2 can facilitate cell proliferation and has been implicated in cancer development [14]. It is also conceivable that all above signaling pathways may be integrated to form networks for cell cycle control via common translation factors (also see below).

## Translation factors are regulated by cellular signaling pathways

Cellular signaling pathways primarily target to translation initiation factors or regulatory factors. Formation/disruption of the eIF4F complex constitutes a major mechanism for controlling cap-dependent translation. The eIF4E-binding proteins (4E-BPs) compete with eIF4G for the same binding site on eIF4E and therefore can prevent eIF4F assembly [15]. The phosphorylation status of 4E-BPs regulates their reversible binding to eIF4E. Hypophosphorylated 4E-BPs bind strongly to eIF4E, whereas the hyperphosphorylation of 4E-BPs (induced by various cellular stresses) prevents binding. The mTOR signaling pathway targets several translation factors, including 4E-BPs, and mTOR-induced phosphorylation of 4E-BPs can contribute to the increase of cap-dependent translation activity during G1 and thus promotes G1 to S progression [16] (Figure 1). eIF4E itself can also be phosphorylated by several different signaling kinases. The level of eIF4E phosphorylation increases in G1 and S phases and is reduced in M phase [17]. Although the impact of eIF4E phosphorylation on translation efficiency has been a matter of debate [18], its phosphorylation via Mnk may result in increased translation of factors involved in cell cycle progression and also has been implicated in cell transformation [19] (Figure 1). Moreover, eIF4E may promote the nuclear export of cyclin D1 mRNA and thus facilitates cell cycle progression [20]. Therefore, Mnk-mediated phosphorylation of eIF4E supports cell proliferation at least in part by promoting synthesis of specific proteins.

The eIF2-GTP/Met/tRNAi ternary complex is another important signaling target at the translation initiation step. Phosphorylation of the eIF2α subunit prevents the recycling of inactive eIF2-GDP to active eIF2-GTP and thus reduces the level of the active ternary complex [21]. A number of protein kinases have been reported to phosphorylate eIF2α, mostly under cell stress conditions. For example, the PKR-like endoplasmic reticulum kinase (PERK) phosphorylates eIF2α in response to endoplasmic reticulum (ER) stress induced by unfolded protein response [22]. In the fission yeast, *Schizosaccharomyces pombe*, that ultraviolet irradiation in G1 phase activates the Gcn2 kinase, which in turn suppresses general translation by phosphorylation of eIF2α and, accordingly, delays S phase entry [23] (Figure 1).

Translation may also be regulated at the elongation step. The elongation factor kinase (eEF2K), also known as a Ca^+2^/calmodulin-dependent kinase, phosphorylates and inactivates eEF2 (Figure 1). eEF2K activity can be modulated by the mTOR signaling pathway or AMPK [24,25]. For example, mTOR complex 1 (mTORC1) inactivates eEF2K by activating the mitotic Cdk1/cyclin B kinase, which may ensure an adequate level of translation during mitosis [25].

## Mechanisms of translational control during cell cycle

**Figure 2:**
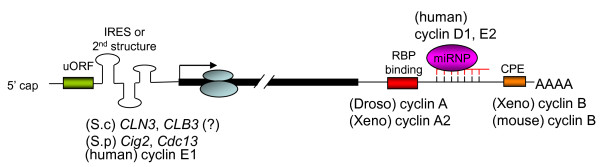
***Cis*-elements of cyclin mRNAs that control their translation**. Translation of mRNAs of budding yeast (S.c) *CLN3*, fission yeast (S.p) *Cig2 *and *Cdc13*, and human cyclin E1 is controlled by uORFs, IRES or structured 5' UTR. The 5' UTR of the budding yeast *CLB3 *mRNA is responsible for its translational control but the underlying mechanism is unclear. Translation of *Drosophila *(Droso) and *Xenopus *(Xeno) type-A cyclin mRNAs is regulated by the Bruno family of RNA binding proteins (RBPs) that bind to the *cis*-elements within their 3' UTR. Expression of vertebrate cyclin B is controlled by CPEB-mediated cytoplasmic polyadenylation and translational control. Expression of two human G1/S cyclins, e.g. cyclins D1 and E2, is regulated by miRNAs.

Cellular signaling pathways induced by changes in environmental conditions often regulate the efficiency of global translation by modulating the activity of essential translation factors. However, some mRNAs undergo specific regulation via *cis*-acting sequences in their 5' or 3' UTRs, such as upstream open reading frames (uORFs) or internal ribosome entry sites (IRESs) in the 5' UTR or binding sites for specific RNA binding proteins or microRNAs (miRNAs) in the 3' UTR [26] (Figure 2). Moreover, a single gene may generate multiple mRNA isoforms with different translation efficiency via use of alternative transcription start sites or polyadenylation sites or alternative splicing. Therefore, translation control is also linked to other steps of post-transcriptional gene expression.

### uORF in the 5' UTR

Short ORFs that are located upstream of or sometimes overlap with the main protein-coding region often disrupt translation of the downstream ORF(s) [26]. An early survey revealed the presence of conserved upstream AUGs in ~20-30% of mammalian genes [27]. A recent study showed that uORFs indeed influences the expression of thousands of mammalian genes [27]. The best-studied example of uORF-mediated translational control is that of the yeast transcription factor GCN4 mRNA [28], which contains four uORFs in its 5' UTR. Translation initiates at uORF1 and then reinitiates at downstream uORFs, leading to inefficient translation of GCN4. During amino acid starvation, the reduced level of functional eIF2 ternary complex retards reinitiation at downstream uORFs, and thus skips these inhibitory uORFs and enhances GCN4 translation. uORFs have been identified in many genes encoding cell growth/survival factors, including bcl-2, c-mos, mdm2, and her-2, and also in genes implicated in the biosynthesis of polyamines, which are important for mitotic spindle formation and chromatin condensation [29,30]. For example, uORF-mediated translation control of an mdm2 mRNA isoform accounts for mdm2 overexpression in some tumors [31]. Moreover, a recent report indicates that disruption of translation initiation within an uORF of the transcription factor C/EBPβ transcript delays S-phase entry [32]. Therefore, the observed high abundance of uORFs in mammalian transcripts suggests a role for uORF-regulated translation in physiology and pathogenesis.

### Secondary structures in the 5' UTR

An early estimation has indicated that ~10% of mRNAs contain atypically long 5' UTRs, and most of these mRNAs encode proteins implicated in cell growth, death or proliferation [33]. Experimental evidence has indicated that longer 5' UTRs account for inefficient translation of certain members or mRNA isoforms of the transforming growth factor β (TGF-β, the tumor suppressor BRCA1 and oncoprotein mdm2 [34-36]. A recent study indicated that two-thirds of human mRNAs harbor a moderate degree of secondary structure in their 5' UTR [37]. Such structures could conceivably impede ribosome scanning or block access of initiation factors to the mRNA and thus reduce translation efficiency. Translation of long or structured 5' UTR-containing mRNAs may require RNA helicases to disrupt RNA duplexes or high-order ribonucleoprotein structures during translation initiation [8]. In fact, several DExD/H-box RNA helicases, including Ded1/DDX3, DHX29 and RNA helicase A, have been demonstrated to facilitate translation initiation of such mRNAs [37-39]. Moreover, we recently reported that translation of cyclin E1 mRNA containing a highly GC-rich 5' UTR is particularly facilitated by the RNA helicase activity of DDX3 [40]. There are probably other RNA helicases that have potential to regulate the translation of perhaps a large number of mRNAs containing structured 5' UTRs.

### IRES-mediated translation

IRESs are RNA structural elements in the 5' UTR that directly recruit ribosomes to the mRNA for translation initiation, thereby bypassing the requirement for the integral eIF4F complex to recognize the 5' cap of the mRNA [41]. In addition to many viral mRNAs, IRESs have been confirmed or predicted in a number of cellular mRNA, many of which encode cell cycle regulators such as ornithine decarboxylase, c-myc, and the Cdk-like kinase p58^PITSLRE ^[42]. Ornithine decarboxylase is involved in polyamine biogenesis, which has an important function for mitosis. The ornithine decarboxylase mRNA undergoes cap-dependent or cap-independent translation initiation at different cell cycle stages; the latter, which is mediated by an IRES, is particularly activated at G2/M [43]. Thus, it is likely that IRES-mediated translation initiation ensures the expression of certain cell cycle or growth factors to allow mitosis to proceed. However, how translation initiation at different types of IRESs engages different combinations of initiation and regulatory factors remains to be deciphered. Moreover, whether previously identified cellular IRESs indeed confer IRES activity has been debated and certainly requires further examination [44].

### Cytoplasmic polyadenylation of the 3' UTR

Cytoplasmic polyadenylation was initially observed in *Xenopus *oocytes where it controls translation of a set of maternal mRNAs such as cyclin B1 and c-mos during oocyte maturation [45,46]. These mRNAs contain the cytoplasmic polyadenylation element (CPE) that is located nearby the polyadenylation signal within the 3' UTR. The CPE-binding proteins (CPEBs) play a central role in regulating translation initiation of CPE-containing mRNAs. While binding to CPE, CPEB recruits the eIF4E-binding protein maskin, which excludes eIF4G, and thus inhibits translation. On the other hand, both the deadenylase PARN and the poly(A) polymerase Gld2 are also a part of the CPEB-containing complex [47]. However, deadenylation by PARN is more efficient and/or Gld2 activity is inhibited so that the poly(A) tail of CPEB-bound mRNAs is shortened in immature oocytes. Oocyte maturation signals induce phosphorylation of CPEB by several different signaling pathways. Phosphorylated CPEB dissociates both maskin and PARN, leading to eIF4F complex formation and predominant polyadenylation, which thus allows reactivation of translation. In early oocytes lacking maskin, the eIF4E-binding protein 4E-T may substitute for maskin to suppress the ability of CPEB to promote polyadenylation [48]. In neurons, neuroguidin acts as a functional analog of maskin to regulate the translation of CPE-containing mRNAs [49]. Therefore, CPE-mediated cytoplasmic polyadenylation and translation plays an important role in early embryonic development and local protein expression in neurons [46].

### RNA-binding protein-mediated translation control

Many RNA-binding proteins control translation in an mRNA-specific manner via binding to *cis*-regulatory elements in UTRs. The mechanisms of such translational regulation have been more extensively studied in *Drosophila*. For example, the *Drosophila *RNA-binding protein Bruno binds to Bruno response elements (BREs) within the 3' UTR of target mRNAs such as *oskar *and recruits the eIF4E binding protein Cup to preclude eIF4G associating with eIF4E and thereby inhibits translation of BRE-containing mRNAs [50]. Moreover, Bruno may induce mRNA oligomerization, which prevents accessibility of ribosomes [51]. Intriguingly, the *Xenopus *Bruno-like protein BrunoL1 binds to the GU/AU-rich BREs of cyclin A2 mRNA, but it promotes, rather than suppresses, translation of cyclin A2 [52]. This observation may not be completely unexpected, however, because these translation regulatory factors may interact with different partners or have different post-translational modifications under different cellular conditions, and therefore differentially affect translation. In addition, mammalian Bruno-like/CELF proteins have been implicated in pathogenesis of the CUG-trinucleotide expansion disease myotonic dystrophy [53]. Therefore, it will be important to elucidate the mechanisms of post-transcriptional control mediated by various RNA-binding proteins, particularly those involved in critical cellular processes or pathogenesis.

### microRNA-mediated translational repression

The single-stranded miRNAs of ~21 nucleotides mediate post-transcriptional gene silencing in the cytoplasm through imperfect base pairing to the 3' UTR of various target mRNAs. miRNAs function in a multi-component complex, termed the RNA-induced silencing complex, to suppress translation and/or cause mRNA degradation [54]. The Argonaute family proteins act as the essential effector in this complex. An estimated >1000 miRNAs are encoded by the human genome and may target ~60% of mRNAs [55]. Some miRNAs are involved in cell cycle control by targeting critical regulatory factors, such as E2F and c-myc, cyclins, Cdks and Cdk inhibitors; misregulation of such a control may contribute to tumorigenesis [56]. Notably, many mRNAs have binding sites for multiple miRNAs. Moreover, the activity of miRNAs can be influenced by the accessibility of their target sites in mRNAs or even modulated by RNA-binding proteins through their interaction with the target mRNAs or the miRNPs. A recent report showed that the RNA-binding protein pumilio binds to the 3' UTR of the Cdk inhibitor p27 mRNA and alters local RNA structure to enhance the accessibility of miR221 and miR-222 [57]. Downregulation of p27 prompts quiescent cells to enter S phase. Furthermore, miRNA activity may even switch from translation repression to translation activation when proliferating cells are induced to enter quiescence [58,59]. Therefore, miRNAs can be versatile translation regulators of the cell cycle.

## Translational control of cyclins

Cyclins form a complex with respective Cdks and activate their kinase activity [60]. Cyclins are essentially divided into the G1/S, S and G2/M classes, and function in a timely manner. Cyclin levels fluctuate during the cell cycle, which is primarily regulated by transcriptional activation and proteolytic destruction. However, accumulating genetic and biochemical evidence has indicated a role for translational control in temporal regulation of cyclin expression (Table 1).

**Table 1:** Translational control of cyclins in the cell cycle

Species	G1/S	S	G2/M
S. cerevisiae	CLN3		CLB3
S. pombe		Cig2	Cdc13
Drosophila		cyclin A	
Xenopus		cyclin A	cyclin B
mammals	cyclin D1, E1, E2		cyclin B

### G1/S cyclins

#### Translational control of CLN3 by a uORF in budding yeast

The temperature-sensitive mutants *cdc33 *or *prt1 *of the yeast *Saccharomyces cerevisiae *show a growth defect in G1 at the non-permissive temperature [61,62]. Because *CDC33 *and *PRT1 *encode eIF4E and an eIF3 subunit, respectively, this observation suggests the importance of translational control in G1 progression. In early G1, a burst expression of the G1 cyclin Cln3 is first observed, which subsequently induces the transcription of downstream cyclins, and is critical for progression through the G1/S boundary. Expression of the *CLN3 *gene is particularly impaired in the *cdc33 *and *prt1 *mutants, which may account for the G1 arrest phenotype of these two mutants [63]. A uORF within the 5' UTR of the *CLN3 *mRNA regulates its expression. Inactivation of this uORF is sufficient to accelerate G1 progression [63]. Moreover, this uORF makes the expression of *CLN3 *sensitive to mTOR signaling [63], suggesting that the level or activity of specific translation initiation factors is important for uORF-mediated translation control. Therefore, such a control may ensure CLN3 expression in prompt response to environmental changes.

#### Translational control of cyclin E1 by DDX3 in mammals

Mammalian DDX3, as its yeast Ded1 homolog, has been implicated in translational control [39,64]. DDX3 may enhance general translation via its interaction with eIF3 and/or promotes the translation of specific mRNAs containing a long or structured 5' UTR by facilitating ribosome scanning during translation initiation. Our recent report showed that knockdown of DDX3 reduces cell growth rate and causes cell cycle arrest in G1 [40]. Screening for target mRNAs of DDX3 revealed cyclin E1 mRNA as a candidate. Cyclin E1 is a G1 cyclin and its complex with Cdk2 triggers S phase entry [60]. Cyclin E1 expression is essentially induced by E2F-mediated transcription in late G1, and its protein level is down-regulated by the ubiquitin-proteasome pathway during S phase. We found that depletion of DDX3 impairs cyclin E1 translation, which provides an explanation for DDX3 deficiency-induced G1 arrest. We also observed that cyclin E1 protein level was diminished in hamster cells upon inactivation of temperature-sensitive DDX3 at a non-permissive temperature. DDX3 facilitates cyclin E1 translation probably by resolving potential secondary structures in its 5' UTR, which has a high GC content (~80%). Notably, the *Xenopus *and *Drosophila *cyclin E1 mRNAs have long 5' UTRs (~500 nt) [40], suggesting that DDX3 has an evolutionarily conserved role in regulating cyclin E1 translation. Moreover, phosphorylation of DDX3 by the mitotic Cdk1/cyclin B kinase is thought to cause translational inhibition during mitosis [65]. Therefore, it would be interesting to know whether DDX3 activity is regulated throughout the cell cycle, which in turn could affect the translation of downstream cell cycle factors.

#### Translational control of cyclin E2 by HCMV miR-US25-1

Human cytomegalovirus (HCMV), a member of the herpesvirus family, encodes at least 11 miRNAs. miR-US25-1 is one of the most highly expressed viral miRNAs during HCMV infection [66]. Interestingly, most miR-US25-1 target genes have been identified as cell cycle regulators, including CCNE2 (cyclin E2) [67]. The miR-US25-1 target sites are usually located within the 5' UTR rather than the 3' UTR of those identified transcripts. miR-US25-1 suppressed the expression of a reporter containing the 5' UTR of cyclin E2, whereas deletion of miR-US25-1 from HCMV up-regulates cyclin E2 expression in the context of viral infection. HCMV infection causes resting cells to re-enter the cell cycle but blocks further progression at the G1/S boundary in order to conduce viral DNA replication. Therefore, perhaps for this purpose, HCMV down-regulates cyclin E2 by expressing miRNAs.

#### Translational control of cyclin D1 by miRNAs

The miR-17/92 cluster of a ~1 kb encodes seven distinct miRNAs within intron 3 of the C13orf25 gene. The observed inverse correlation between the levels of two cluster members, miR-17-5p/20a and cyclin D1, in human breast tumors led to the identification of miR-17-5p/20a as a translational suppressor of cyclin D1 mRNA via binding to its 3' UTR [68]. Accordingly, expression of these two miRNAs could arrest cell cycle at G1 phase and suppress cell proliferation and tumor colony formation. Interestingly, cyclin D1 can transcriptionally up-regulate miR-17/92 expression via binding to its promoter. Therefore, a possible feedback control loop between cyclin D1 and the miR-17/92 cluster may help cells maintain a desired level of cyclin D1. In addition, cyclin D1 was recently identified as a target of miR-193b in melanoma [69]. Because miRNAs can regulate the expression of a myriad of eukaryotic genes and have been implicated in cancer pathogenesis [70], future studies will undoubtedly uncover more cell cycle regulators (including cyclins) as targets of miRNAs. Moreover, accumulating evidence indicating that miRNA activities can be modulated by cellular factors [71]. For example, upon induction of differentiation, the RNA-binding protein RBM4 translocates from the nucleus to the cytoplasm and potentiates the suppressive effect of muscle-specific miR-1 on cyclin D1 translation in muscle cells [72]. Further studies will be required to gain more insights into how the regulation of miRNAs affects cell functions and pathogenesis.

### S cyclins

#### Translational control of B-type cyclins by RNA helicases in fission yeast

The DEAD-box RNA helicase Ded1 of the budding yeast has been implicated in the control of translation initiation [73]. Inactivation of *Ded1 *in *S*. *pombe *particularly impairs the translation of G1/S-specific B-type cyclin *Cig2 *that functions to promote the onset of S phase [74]. *Ded1 *inactivation also inhibited another B-type but G2/M-specific cyclin, *Cdc13*, albeit to a lesser extent than *Cig2*. Another study showed that translation of mRNAs encoding Cdc13 and tyrosine phosphatase Cdc25, which is also a key mitotic regulator, was impaired in the mutants with diminished eIF4A activity [75]. Because the 5' UTR of each of these mRNAs is long or even may contain an uORF(s), their translation thus require RNA helicase activity to facilitate ribosomal scanning or bypass the inhibitory effect of uORFs. These results also suggest that the expression of certain key cell cycle regulators may be tuned at the level of translation.

#### Translational control of cyclin A by Bruno in Drosophila and Xenopus

A-type cyclins play a role during S phase of mitotic cell cycle. During oogenesis, the meiotic cell cycle is arrested initially at prophase I to permit oocyte differentiation and subsequently at metaphase to complete meiosis. Reduction of cyclin A levels early in meiotic prophase is important to maintain the oocyte in meiosis. The translational inhibitor Bruno suppresses translation of cyclin A mRNA by binding to BREs present in its 3' UTR [76], but the underlying mechanism is not clear. Moreover, the poly(A) tail of cyclin A mRNA is shortened by the CCR4-containing deadenylase complex during prophase I arrest, which may affect cyclin A mRNA stability or translation [77,78]. During oocyte maturation, cyclin A1 mRNA resumes translation upon its polyadenylation, which is likely mediated by GLD2 poly(A) polymerase, as well as the loss of Bruno [79]. Therefore, translation suppression of cyclin A1 requires both the timely expression of an RNA-binding protein and polyadenylation control. During organogenesis of *Xenopus*, the Bruno-like protein BrunoL1 is also involved in translational regulation of cyclin A2, which is important for endodermal cell proliferation [52]. In contrast to *Drosophila *Bruno, however, *Xenopus *BrunoL1 activates translation of cyclin A2, although the detailed mechanism remains to be elucidated.

### G2/M cyclins

#### Translational control of cyclin B by cytoplasmic polyadenylation

Cytoplasmic polyadenylation is important for translational control of maternal mRNAs during oogenesis and early embryonic development in many animal species [45,46]. The *Xenopus *cyclin B1 mRNA is a paradigm of cytoplasmic polyadenylation-mediated translational control; its translation is suppressed by shortening of its poly(A) tail in arrested oocytes during the onset of meiotic divisions, and polyadenylation and subsequent translation are induced upon hormone stimulation. Such translational control of cyclin B is also observed during oocyte maturation in mammals [80]. Translation of *Drosophila *maternal cyclin B mRNA is suppressed in the primordial germ cells in order to maintain G2 arrest in the embryonic pole cells [81]. The translational suppressor pumilio binds to the Nanos responsive element in the 3' UTR of cyclin B mRNA and cooperates with Nanos to recruit the CCR4-NOT deadenylase complex [81]. Therefore, mRNA deadenylation also appears to mediate translation regulation of cyclin B1 in the *Drosophila *germline.

#### Translational control of CLB3 in budding yeast during meiosis

Meiosis is a specialized cell division event comprising two chromosome segregation phases, namely the separation of homologous chromosomes in meiosis I and of sister chromatids in meiosis II. Accurate Clb3/CDK regulation is essential for chromosome segregation. During the meiotic cell cycle, three B-type cyclin genes, including *CLB3*, are expressed in meiosis I. However, the activity of Clb3/CDK, which is important for appropriate meiotic chromosome segregation, appears till the onset of meiosis II. Translation of *CLB3 *is likely suppressed in meiosis I and reactivated in meiosis II [82]. The 5' UTR of *CLB3 *mRNA is necessary for its timely restricted translation, but the underlying mechanism remains to be explored.

## Therapeutic implications

The mTOR/PI3K/Akt pathway is involved in the control of both mRNA translation and cell cycle progression and has long been considered a target for anti-cancer therapy [13,14,83,84]. Rapamycin binds to peptidyl-prolyl *cis-trans *isomerase FKBPs and to the FKBP-rapamycin binding domain of mTOR and thus prevents mTORC1 complex formation and subsequent signaling [14]. Therefore, certain rapamycin analogs have been approved for anticancer therapy [85]. However, because mTORC2 is much less sensitive to rapamycin [14], new active-site inhibitors of mTOR have been developed that may completely block the activity of both mTOR-dependent pathways [86]. In addition, because mTOR activity is more effectively suppressed under cell stress conditions such as hypoxia and nutrient deficiency, the use of an AMPK agonist to mimic the effect of energy deprivation also may provide an optional therapeutic strategy [86]. Finally, suppression of cyclin expression can block the cell cycle. Therefore, ERK inhibitors that prevent cyclin D1 expression during mTOR inhibition show a potential for combined therapy by targeting both the mTOR and MAPK pathways [87].

## Conclusions

Because cyclins are central to the control of cell cycle progression, and each acts in a phase-specific manner, cells and embryos must precisely manipulate cyclin levels to promote or suppress the transition between cell cycle phases for different cellular events. Although translational control is not the major mechanism for fluctuation of cyclin levels during the cell cycle, it is important for expression of certain cyclins at specific cell cycle stage, and also provides a means for cells to promptly alter cyclin expression in response to environmental changes. Notably, different translation mechanisms are used to control the expression of different cyclins. Moreover, dysregulation of cyclin expression may cause various diseases involving cell proliferation defects, such as cancer and inflammation. Therefore, a comprehensive understanding of specific mechanisms for the control of cyclin protein expression will provide the basis for developing therapeutic strategy, and perhaps will also potentiate our understanding of stem cell differentiation.

## Conflict of interests

The authors declare that they have no competing interests.

## Authors' contributions

WYT and MCL contributed equally to manuscript preparation. Both authors read and approved the final manuscript.
